# Age-related difference in susceptibility of Apc^*Min*/+^ mice towards the chemopreventive efficacy of dietary aspirin and curcumin

**DOI:** 10.1038/sj.bjc.6600900

**Published:** 2003-04-29

**Authors:** S Perkins, A R Clarke, W Steward, A Gescher

**Affiliations:** 1 Cancer Biomarkers and Prevention Group, Department of Oncology, University of Leicester, Leicester Royal Infirmary, Leicester LE2 7LX, UK; 2 Cardiff School of Biosciences, University of Cardiff, UK

**Keywords:** Apc^*Min*/+^ mice, aspirin, curcumin, chemoprevention

## Abstract

The nonsteroidal anti-inflammatory drug aspirin and the spice curcumin retard adenoma formation when administered long-term to Apc^*Min*/+^ mice, a model of human familial adenomatous polyposis coli. Both agents interfere with cyclooxygenase activity. When aspirin is administered to Apc^*Min*/+^ mice only postweaning, but not before, it is inefficacious, while curcumin given postweaning is active. Here the hypothesis was tested that dietary aspirin (0.05%) or curcumin (0.2%) prevent or delay adenoma formation in offsprings when administered to Apc^*Min*/+^ mothers and up to the end of weaning, but not afterwards. Whereas curcumin was without effect when administered in this way, aspirin reduced numbers of intestinal adenomas by 21%. When aspirin given up to the end of weaning was combined with curcumin administered from the end of weaning for the rest of the animals' lifetime, intestinal adenoma numbers were reduced by 38%. The combination was not superior to intervention postweaning with curcumin alone. These results show that aspirin exerts chemopreventive activity in the Apc^*Min*/+^ mouse during tumour initiation/early promotion, while curcumin is efficacious when given at a later stage of carcinogenic progression. Thus, the results suggest that in this mouse model aspirin and curcumin act during different ‘windows’ of neoplastic development.

It has been estimated that over half of the Western population develops benign adenomatous polyps during its lifetime, and that 10% of these tumours proceed to malignant colorectal carcinoma (Kinzler and Vogelstein, 1996). This realisation has engendered an intense search for efficacious chemopreventive intervention strategies using animal models of premalignant and malignant colorectal cancer. The ‘multiple intestinal neoplasia’ (Apc^*Min*/+^) mouse model of human familial adenomatous polyposis (Moser *et al*, 1990) has been instrumental in the identification of several potential chemopreventive drug candidates, among them nonsteroidal anti-inflammatory drugs (NSAIDs), exemplified by sulindac (Boolbol *et al*, 1996) and aspirin (Mahmoud *et al*, 1998), and the spice curcumin, 1,7-bis(4-hydroxy-3-methoxyphenyl)-1,6-heptadiene-3,5-dione (Mahmoud *et al*, 2000; Perkins *et al*, 2002). Curcumin is the major yellow pigment extracted from turmeric, the powdered rhizome of the herb *Curcuma longa*. Interestingly, the evidence for the chemopreventive efficacy of aspirin in Apc^*Min*/+^ mice, and similar models involving mutant *Apc* is ambiguous. In two studies in Apc^*Min*/+^ mice, aspirin suppressed malignancy (Barnes and Lee, 1998; Mahmoud *et al*, 1998), while in two others in Apc^*Min*/+^ and Apc^*1638N*/+^ mice it failed to show efficacy (Williamson *et al*, 1999; Chiu *et al*, 2000). This discrepancy is probably related to differences in the aspirin regimen used in these studies, a notion borne out by the recent finding that continual exposure of Apc^*Min*/+^ mice to aspirin from the point of conception onwards suppressed tumorigenesis, while exposure during adulthood only did not (Sansom *et al*, 2001). It is not known whether aspirin retains its efficacy in this model when given only during embryogenesis and weaning, without being present in the diet thereafter. Mechanistically curcumin shares with aspirin the ability to interfere with levels of functional cyclooxygenase (COX) enzymes. While aspirin inhibits COX enzyme activity (Vane, 1971), curcumin interferes with the NF*κ*B-mediated activation of COX-2 transcription (Plummer *et al*, 1999). An attractive feature of curcumin is the fact that it fails to elicit detrimental gastrointestinal side effects associated with traditional NSAIDs, such as aspirin. In the study described here, we wished to explore whether dietary aspirin and/or curcumin retard neoplastic development in the Apc^*Min*/+^ mouse when administered *in utero* and during weaning, without being present in the diet thereafter. Aspirin was found to be efficacious when administered in this way, but curcumin was inactive. Therefore, the hypothesis was tested that a combination of aspirin *in utero* and during weaning followed by curcumin postweaning results in additive or synergistic adenoma-suppressing activity, as this regimen might exploit age-related differences in susceptibility of Apc^*Min*/+^ mice to the cancer-delaying effects of these agents.

## MATERIALS AND METHODS

**Figure 1:**
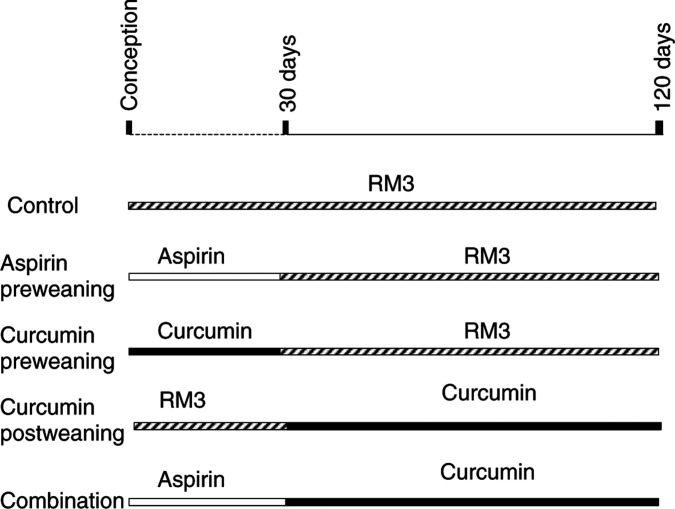
Experimental design for the evaluation of the chemopreventive efficacy of aspirin (0.05%) or curcumin (0.2%) administered in the diet *in utero* and during weaning, or of curcumin from termination of weaning to the end of the experiment, or of the combination of aspirin *in utero* and during weaning followed by curcumin postweaning. RM3 was the control diet. The study was terminated after 120 days. For details of animals and treatments see Materials and Methods.

Experiments in mice were conducted as stipulated by the Animals (Scientific Procedures) Act 1986 Project Licence 80/1250 granted to Leicester University by the UK Home Office, and the experimental design was vetted and approved by the Leicester University Ethical Committee for Animal Experimentation. C57BL/6J male Apc^*Min*/+^ mice and C57Bl/6J female wild-type mice were mated to maintain the Apc^*Min*/+^ breeding colony. Tissue samples were obtained by ear punch and genotyped for Min/+ status by PCR and *Hind*III digest of the product as described previously (Luongo *et al*, 1994). Curcumin and aspirin were purchased from Apin Chemicals (Abingdon, UK) and Sigma (Poole, UK), respectively. The purity of curcumin was verified by HPLC analysis; this material contained 3% desmethoxycurcumin. Aspirin or curcumin was blended into RM3 high protein breeders diet (SDS, Witham, UK), using a mechanical mixer to ensure uniform distribution, which was confirmed by HPLC analysis. Breeding pairs were established and fed RM3 maintenance diet or RM3 containing either 0.05% aspirin, which translates into 75 mg kg^−1^ pd, or 0.2% curcumin, which translates into 300 mg kg^−1^ pd. After 2 weeks, the females were removed and maintained on their respective diets, until the offspring were removed and genotyped at 3 weeks of age. At 30 days, the offspring with the Min/+ phenotype were divided into three intervention groups of eight to 10 animals (Figure 1

): (i) mice that received RM3 control diet, (ii) mice that received either aspirin or curcumin in RM3 diet perinatally and during days 1–30, followed by RM3 diet omitting aspirin/curcumin to the end of the experiment; (iii) mice that received aspirin perinatally and during days 1–30, followed by curcumin in RM3 diet from weaning to the end of the experiment. The early administration regimen will be referred to in the following as ‘*in utero* and during weaning’, the late regime as ‘postweaning’. An experiment, in which mice received curcumin postweaning to the end of the experiment, has been performed previously in this laboratory (Perkins *et al*, 2002) and was not repeated here to reduce animal usage. At 120 days, mice were killed by cardiac exsanguination under terminal halothane anaesthesia. The gastrointestinal tract was removed, and multiplicity, location and size of adenomas were recorded as described previously (Perkins *et al*, 2002). Adenoma numbers values were subjected to statistical evaluation by ANOVA using Excel and Minitab software packages (Microsoft Windows, 1997). Statistical significance (*P*<0.05) was established by *post hoc* Tukey's pairwise comparison. The haematocrit, the percentage of blood volume occupied by packed erythrocytes, was determined as described previously (Strumia *et al*, 1954) using blood samples collected and drawn by capillary force into heparinised microhaematocrit tubes (75 mm, Richardson's, Leicester, UK).

## RESULTS AND DISCUSSION

**Figure 2:**
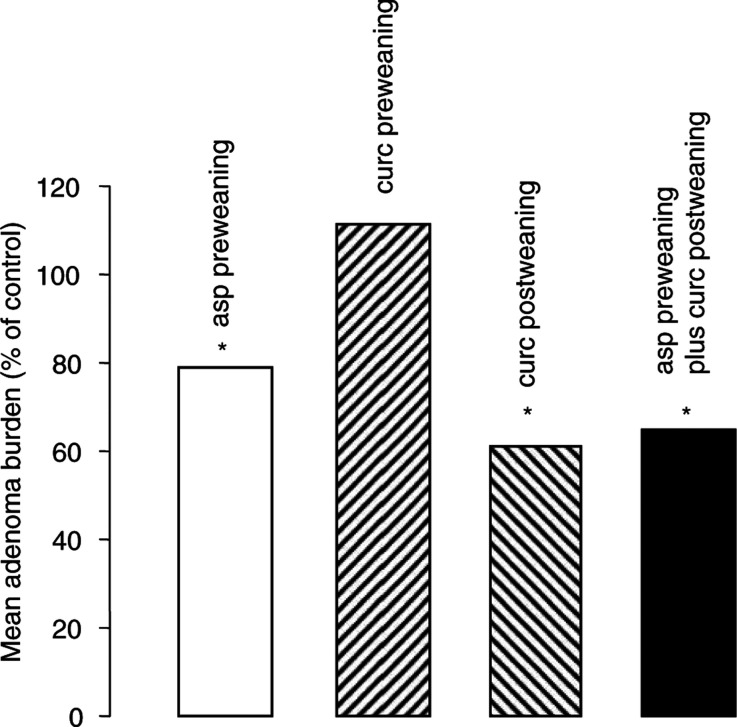
Effect on adenoma burden in the small intestine of Apc^*Min*/+^ mice of aspirin (‘asp’, 0.05% in the diet, open bar) or curcumin (‘curc’, 0.2% in the diet) administered *in utero* and during weaning (‘preweaning’, bar striped diagonally bottom left to top right), or of curcumin administered postweaning to the end of the lifetime (‘postweaning’, bar striped diagonally top left to bottom right), or of the combination of aspirin *in utero* and during weaning followed by curcumin postweaning (black bar). Adenoma burden is expressed as percentage of number of adenomas in untreated mice, the number of mice used per group was eight to 10. The value for the effect of curcumin postweaning (bar striped diagonally top left to bottom right) was obtained previously (Perkins *et al*, 2002) and has been included for comparison; this experiment was not repeated here to minimise animal usage. The results originate from three separate experiments, and number of intestinal adenomas in the control (untreated) groups were as follows: experiment described by open and black bars: 132±12, experiment described by bar diagonally striped bottom left to top right: 117±13, experiment described by bar striped diagonally top left to bottom right: 115±12. The s.d.s of adenoma number values for the different interventions are 12% of the mean, or smaller. Asterisk indicates that the number of adenomas is significantly different from that in control (untreated) animals (*P*<0.05). For details of animals and treatments and statistical evaluation, see Materials and Methods.

Administration of aspirin (0.05%) *in utero* and during weaning in Apc^*Min*/+^ mice and maintaining mice on aspirin-free diet thereafter, reduced tumour burden in the small intestine by 21% (Figure 2

). This result is consistent with the notion that the majority of adenomas in Apc^*Min*/+^ mice are fixed already either *in utero* or perinatally just after birth (Shoemaker *et al*, 1995; Ritland and Gendler, 1999) It suggests, for the first time, that interference with tumour initiation and/or early promotion in Apc^*Min*/+^ mice can have a long-term beneficial consequence, even if the chemopreventive stimulus is discontinued postweaning. A similar reduction was observed in the colon, however overall colonic adenoma burden was so low that the difference between exposed and unexposed mice was not significant (result not shown). The modest but significant reduction of intestinal adenoma burden by aspirin is consistent with previous work according to which long-term dietary administration of aspirin from conception onwards increased the survival of Apc^*Min*/+^ mice, while exposure during adulthood only did not (Sansom *et al*, 2001). The failure of aspirin to attenuate neoplastic development in Apc^*Min*/+^ mice, when administered postweaning only, has been demonstrated in at least three other studies (Williamson *et al*, 1999; Chiu *et al*, 2000; Reuter *et al*, 2002). In contrast, there are reports that document convincingly the ability of two NSAIDs other than aspirin, piroxicam (Ritland and Gendler, 1999) and celecoxib (Jacoby *et al*, 2000b), to decrease the number of established polyps and to prevent the development of nascent ones, when they are administered at a late stage during the lifetime of Apc^*Min*/+^ mice.

**Figure 3:**
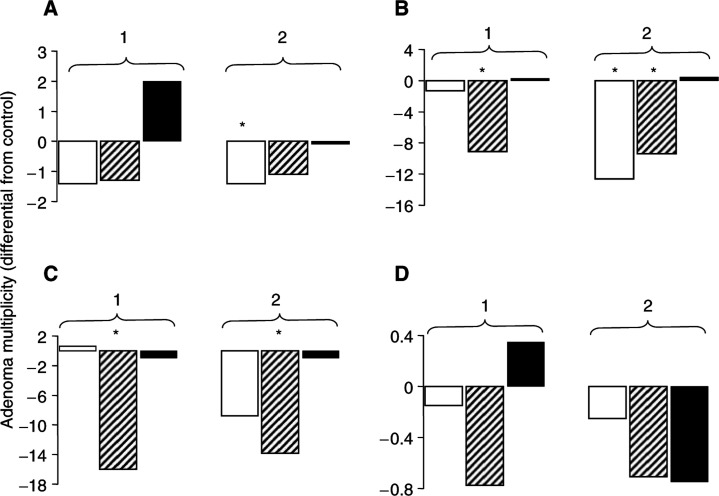
Effect of dietary aspirin (0.05%) administered *in utero* and during weaning (bars 1) or of the combination (bars 2) of aspirin, given as above, with dietary curcumin (0.2%), administered postweaning to the end of the experiment, on multiplicity of small (<1 mm diameter, open bars) medium size (1–3 mm, hatched bars) or large (>3 mm, closed bars) adenomas in the proximal (**A**), middle (**B**), distal (**C**) or colonic (**D**) sections of the intestine of Apc^*Min*/+^ mice. Results are expressed as mean number of adenomas over or below mean adenoma numbers in control (untreated) Apc^*Min*/+^ mice. Number of mice per group was eight to 10. Asterisk indicates that the number of adenomas in that segment was significantly different from that in the respective segment in control animals (*P*<0.05). For details of animals and treatments and statistical evaluation see, Materials and Methods.

Detailed analysis of the results obtained for aspirin reveals that administration *in utero* and during weaning reduced the number of middle-sized adenomas, those of 1–3 mm diameter, in both the middle and distal regions of the small intestine (Figure 3

). The decrease in tumour size intimates that aspirin delays adenoma development, rather then totally suppressing the emergence of a subset of adenomas. The efficacy of aspirin when it is administered *in utero* and during weaning only suggests that in the Apc^*Min*/+^ mouse there is a ‘window of opportunity’ for preventive intervention using aspirin, and this window occurs in very young mice. A similar window of susceptibility allowing regulation of tumour development in Apc^*Min*/+^ mice has been suggested by results of experiments in which the effect of the carcinogen *N*-ethyl-*N*-nitrososurea on the formation of crypts and adenomas was studied (Shoemaker *et al*, 1995).

In contrast to aspirin, dietary curcumin (0.2%) administered *in utero* and during weaning only, failed to affect adenoma number (Figure 2). This finding suggests that high preventive efficacy at the stage of tumour initiation/early promotion is not a generic feature of all agents that target COX enzymes. In contrast, curcumin administered later, that is, from the end of weaning for the lifetime, reduced intestinal adenoma burden in Apc^*Min*/+^ mice by 39%, compared to untreated mice (Figure 2, reference Perkins *et al*, 2002).

These results warrant interpretation in terms of our knowledge of the pharmacokinetics of aspirin and curcumin. Aspirin is efficiently absorbed, rapidly distributed and swiftly hydrolysed in the biophase to salicylate, which in turn is eliminated via the kidneys and/or undergoes phase II drug metabolism (Needs and Brooks, 1985). Furthermore, salicylate generated by hydrolysis of aspirin reaches breast milk readily (Findlay *et al*, 1981). When administered to the mother, salicylates are rapidly transferred to the fetus (Schoenfeld *et al*, 1992). As aspirin has a short half-life, only a small amount of unmetabolised drug reaches the fetus, which is therefore exposed mainly to its metabolite salicylate. Compared to the adult organism, the fetus has reduced abilities of salicylate plasma protein binding, biotransformation and drug elimination. Therefore, fetusses and newborns whose mothers received aspirin before delivery may have plasma concentrations of free salicylate up to four times higher than those of their mothers (Schoenfeld *et al*, 1992). The finding that aspirin exerted chemopreventive efficacy when administered *in utero* and during weaning is consistent with these pharmacokinetic considerations, in that efficacy was probably the consequence of efficacious levels of salicylate in the mother's milk and the embryonic blood and tissues. In contrast, the absorption of curcumin is poor and its systemic availability is extremely low in all species in which it has thus far been tested (Ireson *et al*, 2001). Therefore, when curcumin was administered *in utero* and during weaning in Apc^*Min*/+^ mice, levels of drug which reached the maternal blood and milk and the fetal organism were conceivably insufficient to elicit chemopreventive efficacy.

Sequential administration in Apc^*Min*/+^ mice of firstly aspirin *in utero* and during weaning and secondly curcumin given postweaning for the remainder of the animals' lifetime decreased mean tumour burden slightly, but not significantly, more than the aspirin-only regimen (Figure 2). The extent of adenoma reduction by the combination was also not superior to intervention with curcumin alone administered postweaning (Figure 2). Analysis of tumour distribution (Figure 3) shows that sequential intervention with aspirin followed by curcumin significantly reduced the number of small adenomas in the proximal and distal regions and of middle-sized adenomas in the middle and distal regions, which is comparable to the efficacy characteristics of curcumin alone (Perkins *et al*, 2002).

Even though aspirin and curcumin are considered to act via similar modes of action by decreasing levels of active COX enzymes, there are clear differences between them as reflected by the age-related discrepancy in susceptibility of Apc^*Min*/+^ mice towards drug activity. On the one hand, aspirin and curcumin seem to exert optimal adenoma-retarding activity at different stages of the lifetime of Apc^*Min*/+^ mice, aspirin early and curcumin late; on the other hand, we failed to observe additivity or synergy when both agents were administered sequentially. Together these findings are consistent with the notion that in this mouse model aspirin and curcumin exert their activities probably on the same cells, but within different ‘developmental windows’.

The administration regimens involving aspirin *in utero* and during weaning alone or in combination prior to curcumin had no detrimental effect on propensity towards gastrointestinal bleeding, as reflected by the haematocrit (results not shown), nor did they cause gastric erosion and loss of mucosal integrity, as adjudged by macroscopic inspection. These side effects are often associated with long-term administration of NSAIDs.

What is the corollary of these results for cancer chemoprevention in humans? *In utero* administration of drugs is obviously not a feasible intervention strategy in humans. Nevertheless, the proof of principle study described here allows the conclusion that that there seem to be different ‘windows of susceptibility’ during preneoplastic and neoplastic development, in which agents exert their preventive activities differentially, at least in the Apc^*Min*+^ mouse. Applied to humans this realisation adds another layer of complexity to the design optimisation of intervention trials using combinations of agents. Multiagent chemoprevention strategies in Apc^*Min*/+^ mice which have been efficacious are combinations of piroxicam with difluoromethylornithine (Jacoby *et al*, 2000a), and of sulindac with the epidermal growth factor receptor kinase inhibitor EKI-569 (Torrance *et al*, 2000) or with tea polyphenols (Orner *et al*, 2002). A better delineation of the developmental window which permits optimal efficacy for each agent might help to improve the use of combination chemoprevention strategies in humans.
